# 2-Dimensional Speckle Tracking Echocardiography predicts severe coronary artery disease in women with normal left ventricular function: a case-control study

**DOI:** 10.1186/s12872-017-0656-5

**Published:** 2017-08-24

**Authors:** Ryan T. Hubbard, Maria C. Arciniegas Calle, Sergio Barros-Gomes, Joyce A. Kukuzke, Patricia A. Pellikka, Rajiv Gulati, Hector R. Villarraga

**Affiliations:** 1 Department of Physical Medicine and Rehabilitation, Mayo Clinic, Rochester, MN USA; 2 Department of Cardiovascular Diseases, Mayo Clinic, 200 First St SW, Rochester, MN 55905 USA; 3 Department of Orthopedic Research, Mayo Clinic, Rochester, MN USA

**Keywords:** Coronary artery disease in women, Dyssynchrony, Normal ejection fraction, 2D-STE

## Abstract

**Background:**

Women who have coronary artery disease (CAD) often present with atypical symptoms that may lead to misdiagnosis. We assessed strain, systolic strain rate and left ventricular dyssynchrony with 2- dimensional- speckle tracking echocardiography to evaluate its use as a non-invasive method for detecting CAD in women with normal ejection fraction compared with healthy women controls with a normal angiogram.

**Methods:**

We included 35 women with CAD confirmed by coronary angiography and a positive exercise stress echocardiography and 35 women in a control group with a low pretest probability of CAD, normal angiogram and a normal stress echocardiography with normal EF.

**Results:**

Statistically significant 2D-STE findings for the CAD vs control groups were as follows for the mean of: global circumferential strain (CS) (−19.4% vs −22.4%, *P* = .02); global radial S (49% vs 34%, *P* = .03); global radial SR (2.4 s^−1^ vs 1.9 s^−1^, *P* = .05); global longitudinal LV S (GLS) (−14.3% vs −17.2%, *P* < .001). For mechanical dyssynchrony, SD of the GLS time-to-peak (TTP) was computed (99 vs 33 ms, *P* < .001). The receiver operating characteristic and area under the curve (AUC) were calculated. A cutoff value of 45 ms for 1 SD of the longitudinal S TTP had 97% sensitivity and 89% specificity (AUC, 0.96). GLS cutoff value of −15.87% had 71% sensitivity and 74% specificity; AUC, 0.74 in differentiating CAD and control groups. The combined GLS, CS, and SD of the longitudinal S TTP had an AUC of 0.96 (sensitivity 97%, specificity 86%). Interclass correlations of the GLS segment and GLS TTP measurements were 0.49 (95% CI, 0.227-0.868) and 0.74 (95% CI, 0.277-0.926), respectively.

**Conclusion:**

In women with a normal echocardiogram and LVEF, CAD can be identified by dyssynchrony and abnormal strain values, as evidenced by 2D-STE.

## Background

Cardiovascular disease is the leading cause of morbidity and mortality throughout most of the world today, representing 31% of all deaths [1]. Significant advances have been made in the diagnosis and treatment of cardiovascular disease, but most have been tailored to the recognition and treatment of the disease in men [2]. Substantially less research has been done to study the growing issue of the disease in women [3]. Women tend to present with atypical symptoms and experience coronary artery disease (CAD) later in life than men. They also have a higher incidence of morbidity and mortality when CAD does occur, including myocardial infarction and sudden death [4]. Women also have a different atherosclerotic profile than men, primarily composed of microvascular disease without substantial obstructive CAD on imaging [5]. Because of this finding, it has been suggested that CAD in women be referred to as “female specific ischemic heart disease” [5–7].

Echocardiography is an important noninvasive method for assessing cardiovascular function and mechanics [8]. Two-dimensional–speckle tracking echocardiography (2D-STE) has proven to be an important recent advance [8, 9]. 2D-STE software tracks groups of intramyocardial speckles to derive myocardial deformation in 3 imaging planes, allowing measurement of parameters such as strain (S) and systolic strain rate (SRs) of the myocardium [10]. Measurements of S and SRs are more sensitive than standard echocardiographic parameters for assessing left ventricular (LV) function of many clinical conditions, including cardiomyopathy and CAD [11–14]. 2D-STE has been validated as an accurate, angle-independent, noninvasive method for evaluating cardiac mechanical function [15]. Furthermore, STE by velocity vector imaging (VVI) has been confirmed as an accurate method to measure S and SRs and to quantify myocardial function regionally and globally [16].

The aim of this study was to assess 2D-STE parameters of S, SRs, and time-to-peak (TTP) measurements, as well as LV dyssynchrony in healthy women to define normal values and then to compare these to values of women with known CAD. By so doing, we hoped to demonstrate that 2D-STE can be used to predict CAD in women with a normal ejection fraction (EF).

## Methods

### Study population

We reviewed electronic health records of women who underwent an exercise stress echocardiography, followed by cardiac angiography that showed severe CAD. Severe CAD was classified as stenosis of more than 50% in 1 or more vessels and was further sub-classified depending on the number of affected vessels [17]. This classification was based on current practice guidelines which place patients with higher than 50% stenosis at high risk for adverse cardiac events [18–21]. All tests were performed at Mayo Clinic from January 1, 2006, through December 31, 2006, and coronary angiography had to be completed within 6 months of the treadmill test. Patients were excluded if they had a resting LVEF less than 50%, a contrast computed tomography scan, atrial fibrillation, more than moderate valvular disease, or studies with poor image quality. The control group comprised women with a low probability of CAD, ie, normal EF and a normal stress echocardiogram and coronary angiogram performed at Mayo Clinic from 2009 through 2010. The Mayo Clinic Institutional Review Board approved the study, and all patients provided written consent to allow review of their electronic clinical records and images.

### Standard echocardiographic examination

Echocardiographic imaging was performed by a registered diagnostic cardiac sonographer using a standardized protocol in the Mayo Clinic echocardiography laboratory. Three commercial echocardiographic systems were used: 1) Sequoia C512 (Siemens AG, Munich, Germany) with a 4 V1 transducer (1-4 MHz); 2) Vivid 7 (General Electric Co, Fairfield, Connecticut) with an M4S transducer (1.5-4.3 MHz); and 3) iE33 (Royal Philips Electronics, Amsterdam, The Netherlands) with an S5-1 transducer (1-5 MHz). The Sequoia C512 was used for all women in the control group. Images were obtained at a mean (SD) frame rate of 41 (5) MHz in the CAD group.

### Speckle tracking imaging

**Fig. 1:**
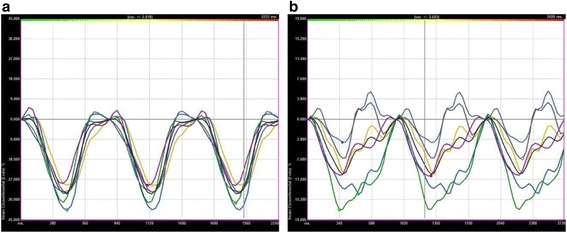
Left ventricular deformation. **a** Normal pattern left ventricular deformation. **b** Coronary artery disease. These images were exported and then analyzed offline with *syngo* VVI (velocity vector imaging) software

Three-beat cine-loop clips were selected from the parasternal short-axis views at the papillary muscle level and from 3 apical views (2-chamber, 3-chamber, and 4-chamber). These images were exported and then analyzed offline with *syngo* VVI software (Siemens Medical Solutions USA, Inc., Malvern, Pennsylvania). The process began with manual endocardial to mid-wall tracing of a single frame at end systole by a point-click approach, with a region of interest that covers at least 90% of the myocardial wall thickness. The periodic displacement of the tracing was automatically tracked in subsequent frames. Tissue velocity was determined by the software, according to a shift of the points divided by time between B-mode frames. The software automatically calculated S and SRs from the velocity. The TTP of S and SRs was measured automatically by the software, with the beginning of the QRS complex as a reference. The SD of longitudinal S for 16 segments was calculated. LV dyssynchrony was defined according to the method proposed by Yu et al. [22], described below. VVI data were exported into an Excel 2003 spreadsheet (Microsoft Corp, Redmond, Washington) for further analysis (Fig. 1).

### Reproducibility

Interobserver variability was assessed in 10 randomly selected patients for whom 2D-STE examinations were independently performed by 2 investigators (S.B.G., J.A.K.) 4 to 8 weeks after the initial analysis. The intra-subject reproducibility of data was evaluated with the intra-class correlation coefficient, which measured the strength of the association between the measured parameters [23–25].

### Statistical analysis

Data are presented as mean (SD) for continuous variables and as percentages for categorical variables. Differentiation between the CAD and control groups was performed with univariate and multivariate logistic regression. Each global and segmental S and SRs value was assessed as a predictor of the CAD group in a model. Diagnostic accuracy of S and SRs values was evaluated using receiver operating characteristic (ROC) analysis. We used the concordance correlation coefficient (CCC) for interobserver variability and calculated the absolute difference and the percentage difference between the 2 observers. A *P* value of less than .05 was considered statistically significant. JMP 9.0.1 statistical software (SAS Institute Inc., Cary, North Carolina) was used to perform the statistical analysis.

## Results

### Participant characteristics

The mean (SD) values for the CAD and control groups (35 women in each group), respectively, were as follows: age, 70 (8) vs 35 (13) years (*P* < .001); body mass index, 27 (5) vs 26 (7) kg/m^2^ (*P* = .23); heart rate, 76 (12) vs 75 (11) beats per minute (*P* = .94); systolic blood pressure, 137 (19) vs 110 (15) mm Hg (*P* < .001); diastolic blood pressure, 74 (10) vs 65 (10) mm Hg (*P* = .004); PR interval, 165 (21) vs 143 (17) ms (*P* < .001); and QRS duration, 84 (10) vs 88 (12) ms (*P* = .17). CAD was classified as follows: mild disease (<50% occlusion), 8.6% (3 patients); and severe 1-vessel disease, 31.4% (11 patients); 2-vessel disease, 31.4% (11 patients); and 3-vessel disease, 29% (10 patients). The most common indications for further diagnostic studies in the CAD group were fatigue and substantial comorbidities or risk factors for CAD. Only 3% of controls had comorbidities, such as hypertension and hyperlipidemia. Other clinical characteristics of the groups are shown in Table 1.

**Table 1:** Group characteristics

	Group^a^		
Characteristic	Coronary artery disease	Control	*P*
Age, mean (SD), y	70 (8)	35 (13)	<0.001
BMI, mean (SD)	27 (5)	26 (7)	0.23
SBP, mean (SD)	137 (19)	110 (15)	<0.001
DBP, mean (SD)	74 (10)	65 (10)	=0.004
HR, mean (SD)	76 (12)	75 (11)	0.94
PR interval, mean (SD)	165 (21)	143 (17)	<0.001
QRS duration, mean (SD)	84 (10)	88 (12)	0.17
Hypertension	71	3	<0.001
Hyperlipidemia	69	3	<0.001
Dyspnea	100	0	<0.001
Fatigue	23	0	<0.001
Diabetes mellitus	9	0	<0.001
Smoker, ever	26	3	<0.001

Abbreviations: *BMI* body mass index, *DBP* diastolic blood pressure, *HR* heart rate, *SBP* systolic blood pressure

^a^% unless otherwise noted

### Standard echocardiographic data

The mean (SD) 2D echocardiographic findings for the CAD group vs the control group, respectively, were as follows: EF, 60%(5) vs 63% (4) (*P* = .02); LV systolic dimension, 29 mm (5) vs 30 mm (3) (*P* = .52); early mitral inflow velocity (E) wave, 0.76 (0.25) m/s vs 0.84 (0.23) m/s (*P* = .16); and early diastolic mitral annular tissue velocity (e′), 0.09 (0.14) m/s vs 0.12 (0.03) m/s; (*P* < .001). Variables with higher (mean [SD]) values in the CAD group were the E/e′ ratio (11.9 [1] vs 7.8 [1]; *P* = .002); peak velocity of late transmitral flow (0.89 [0.19] m/s vs 0.54 [0.22] m/s; *P*<.001); tricuspid regurgitation velocity (2.73 [0.76] vs 2.22 [0.25]; *P* = .002); Right ventricular systolic pressure (33.5 [8] vs 25.0 [6]; *P* < .001); left atrial volume index (29 [10] vs 26 [13]; *P* = .01); and left ventricular diastolic dimension (46.4 [6] mm vs 46.3 [4] mm; *P* = .95).

### Global longitudinal, circumferential and radial S and SRs

**Fig. 2:**
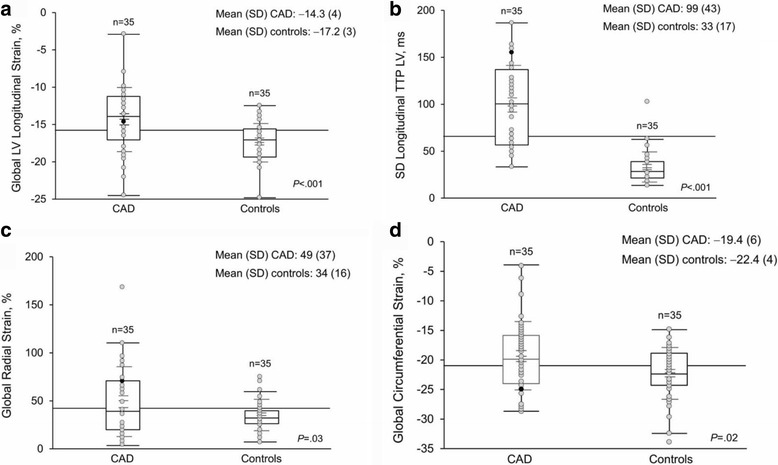
Differences between the coronary artery disease and control groups. **a** Global LV longitudinal strain (*P* < .001). **b** dyssynchrony (*P* < .001). **c** Global radial S (*P* = .03). **d** Global circumferential strain (*P* = .02). LV indicates left ventricle; TTP, time-to-peak

Values in the CAD group vs the control group (mean [SD]) were as follows: global longitudinal strain (GLS) (average, 16 segments) (−14.3% [4] vs −17.2% [3]; *P* < .001); global longitudinal SRs (−0.92 s^−1^ [0.2] vs −0.99 s^−1^ [0.1]; *P* = .07); global circumferential strain (CS) (−19.4% [6] vs −22.4% [4]; *P* = .02) and global circumferential SRs (−1.3 s^−1^ [0.4] vs −1.4 s^−1^ [0.3]; *P* = .16) Values (mean [SD]) were higher in the CAD group than in the control group for the following: global radial S (49% [37] vs 34% [16]; *P* = .03); global radial SRs (2.4 s^−1^ [1.5] vs 1.9 s^−1^ [0.7]; *P* = .05); and SD global longitudinal LV S TTP (99 ms [43] vs 33 ms [17]; *P* < .001) (Fig. 2, Table 2). The differences in global radial S, global radial SR, and 1 SD of the GLS TTP remained significant after adjustment for heart rate, blood pressure, LVEF, and body mass index in a multivariate model (odds ratio [OR], 5.4 [95% CI, 1.9-54.0]; *P*<.001), as well as for global longitudinal SRs (OR, 10 [95% CI, 2.5-203.9]; *P* < .001). In addition, we acquired data for the left ventricular mass index (LVMi) in order to classify patients with a value of >95 g/m2 as having left ventricular hypertrophy. According to this classification, six patients in the CAD group had left ventricular hypertrophy. However, the mean LVMi was 92 g/m^2^ (SD 31 g/m^2^) in the CAD group and 74 g/m^2^ (SD 15 g/m^2^). When we added LVMi to the multivariate model, dyssynchrony remained significant with a *p* value <0.01. Age proved to be a strong predictor (OR 1.25 [95% CI, 1.14-1.49]; *P* < .001); however, dyssynchrony continued to remain significant after adjusting for this variable. The cutoff value of −15.87% for GLS had 71% sensitivity and 74% specificity in differentiating the CAD group from the control group, with a ROC area under the curve (AUC) of 0.74 Table 3). The cutoff value of −21.31% for CS had 60% sensitivity and 60% specificity for differentiating the CAD group from the control group, with an AUC of 0.64 (Table 3).

**Table 2:** Longitudinal and circumferential strain, systolic strain rate, and dyssynchrony

	Group^a^		
Variables	Coronary artery disease, Mean (SD)	Control, Mean (SD)	*P* Value
Global longitudinal LV strain, %	−14.3 (4)	−17.2 (3)	<.001
Global longitudinal systolic strain rate, s^−1^	−0.92 (0.2)	−0.99 (0.1)	0.07
Global longitudinal LV strain TTP, ms	399 (65)	381 (46)	<.001
1SD Global longitudinal strain TTP, ms	99 (43)	33 (17)	<.001
Global circumferential strain, %	−19.4 (6)	−22.4 (4)	0.02
Global circumferential systolic strain rate, s^−1^	−1.3 (0.4)	−1.4 (0.3)	0.16
Global radial strain, %	49 (37)	34 (16)	0.03
Global radial systolic strain rate, s^−1^	2.4 (1.5)	1.9 (0.7)	0.05

Abbreviations: *LV* left ventricular, *TTP* time-to-peak

^a^Mean (SD) unless otherwise indicated

**Table 3:** Data for receiver operating characteristic curves

Measurement	Cutoff value	Sensitivity, %	Specificity, %	AUC
Global longitudinal strain, %	−15.87	71	74	0.74
1 SD LV longitudinal TTP, ms	45	97	89	0.96
2 SD longitudinal strain TTP, ms	90	97	89	0.96
Global circumferential strain, %	−21.31	60	60	0.64
Combined plot (global longitudinal strain, circumferential strain, SD longitudinal TTP LV)	NA	97	86	0.96

Abbreviations: *AUC* area under the curve, *LV* left ventricular, *NA* not applicable, *TTP* time-to-peak

### LV dyssynchrony

**Fig. 3:**
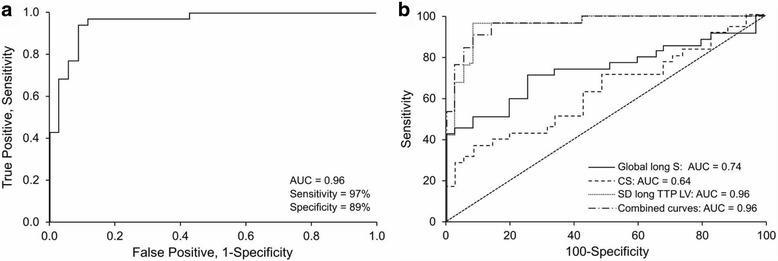
**a** By using a cutoff value of 45 ms, for dyssynchrony, we were able to detect CAD in women with a sensitivity of 97% and specificity of 89%, with an AUC of 0.96. **b** By combining global longitudinal strain, CS and longitudinal dyssynchrony, the AUC was 0.96, with 97% sensitivity and 86% specificity. AUC indicates area under the curve; CAD, coronary artery disease; CS, circumferential strain; long, longitudinal; LV, left ventricular; lin1 (3), combination of global longitudinal S, CS, and SD long TTP LV; S, strain; TTP, time-to-peak

To assess mechanical dyssynchrony, the SD of GLS TTP was computed for the CAD and control groups, respectively (99 [43] ms vs 33 [17] ms; *P* < .001) (Table 2). To standardize for these differences, we followed the method described by Yu et al. (22). In brief, the mean of the SD plus 2 SDs for the control group were calculated from the GLS TTP. With this method, 65.87 ms was the cutoff value; 1 patient (3%) in the control group and 23 (66%) in the CAD group were above that value (*P* < .001). Values for longitudinal dyssynchrony for the control group and CAD group were significantly different (*P* < .001) (Fig. 2b). In addition, by using a cutoff of 45 ms for 1 SD LV longitudinal TTP, we were able to detect CAD in women with a sensitivity of 97% and specificity of 89%, with an AUC of 0.96 (Fig. 3a, Table 3). When we combined GLS, CS, and the SD of the LV longitudinal TTP, the sensitivity to detect CAD was 97% and specificity was 86% (Fig. 3b, Table 3).

### Feasibility and reproducibility of S and systolic TTP measurements

STE tracking accuracy was assessed visually by one of the authors (R.T.H.). Measurements of S and TTP with acceptable tracking were feasible in 94.3% of the segments of the CAD group and in 97.1% of the segments in the control group. The intraobserver mean (SD) differences in longitudinal S and TTP variability were 8% (4%) and 7% (6%), respectively. The mean differences in CS and SRs variability were 6% (5%) and 10% (18%), respectively. The interobserver mean (SD) differences in variability for longitudinal S and SRs were 9% (8%) and 9% (5%), respectively. The CCC was calculated from 10 patients to compare reproducibility between the 2 examiners (J.A.K., S.B.G.). The CCC values for global longitudinal S and longitudinal S TTP were 0.922 (95% CI, 0.723-0.979) and 0.79 (95% CI, 0.446-0.931), respectively. Analysis was done using MedCalc software, version 14.10.2 (MedCalc, Ostend, Belgium).

## Discussion

We performed a retrospective study to evaluate the clinical and echocardiographic variables of patients with documented CAD and compared them to patients with no CAD [26]. To our knowledge, this is the first study to measure S, SRs, TTP, and dyssynchrony in a population of women with known CAD. Our main findings were a significant reduction in longitudinal and CS and dyssynchrony in the CAD group compared with the control group. These findings were consistent in all longitudinal views (2-, 3-, and 4-chamber) and globally. The differences remained significant after adjusting for other clinical variables, such as heart rate, LVEF, left ventricular hypertrophy, blood pressure, body mass index, body surface area, and hyperlipidemia in a multivariate model analysis.

In addition, we were able to predict CAD using dyssynchrony with 97% sensitivity and 89% specificity and an AUC, 0.96. The results clearly show that GLS, longitudinal TTP, and CS were reduced, even though right ventricular S remained normal; this finding suggests that longitudinal S and TTP impairment occur early in response to insults that might result from CAD.

Other noninvasive methods have been used to evaluate the risk for CAD in women, including measurements of high-sensitivity C-reactive protein (HsCRP) and coronary artery calcium scoring [27]. The role of HsCRP has not been fully elucidated in studies to date. However, in women with metabolic syndrome, high levels of HsCRP have been found to correlate with a doubled risk of future cardiovascular events when women with this condition are compared with women who have metabolic syndrome but low levels of HsCRP [27]. In the MESA trial (Multi-Ethnic Study of Atherosclerosis), the coronary artery calcium score was shown to be a more sensitive risk-prediction tool than even the Framingham risk score for congestive heart disease and CAD in low-risk populations, demonstrating that the presence of any coronary calcium was associated with a 6-fold increased risk of CAD [28]. Although more studies of HsCRP and coronary artery calcium are needed, these methods represent advances being made in the noninvasive detection and risk assessment of CAD in women [29]. The MESA study particularly shows a relative inadequacy of the Framingham risk score for predicting CAD in women and highlights the need for better assessment of women in the low-risk category. We present our study as an adjunctive approach to the novel assessment of CAD risk [30].

CAD is an increasing concern in women, and as described earlier, more novel methods are needed to better predict its presence and severity. As women present to the clinic with nonspecific symptoms of CAD, clinicians need feasible diagnostic procedures to assess risk and likelihood of disease in order to better approach patient care. The findings of our study have the potential to greatly impact the advances in early detection and risk stratification of CAD in women, which could lead to preservation of cardiac function and a decrease in overall morbidity and mortality [22].

On the basis of our research, we believe that S and dyssynchrony parameters, measured by 2D-STE, could predict CAD in a population of symptomatic and asymptomatic women with normal EF. Our current study validates the need for a larger longitudinal study in women to determine the initial decline in S and systolic SR and to determine how this noninvasive method can project the overall decline in cardiac function and the early signs of ischemic insult. Such noninvasive measurements could allow a more aggressive approach for patients with these characteristics.

### Limitations

This study was retrospective and limited by its small size. This resulted from the need to have a normal angiogram in all the controls. Our population was older because younger women were less likely to have an indication for stress echocardiography. Furthermore, it was challenging to find patients for the control group in the same age range as the study population because of the higher prevalence of cardiac issues in patients older than 70 years. Although our study population comprised older women with known CAD, which placed them in the high-risk Framingham category, our results confirm another useful, noninvasive method for stratifying patients who may have equivocal risk factors for CAD to better determine the need for aggressive medical management.

## Conclusion

LV dyssynchrony measurements and longitudinal, circumferential, and radial S shown on 2D-STE can predict severe CAD in women with normal LVEF. This new technique should be incorporated into the diagnostic algorithm when there is suspicion that a woman might have CAD.

## Abbreviations

2D-STE: 2-dimensional–speckle tracking echocardiography
AUC: Area under the curve
CAD: Coronary artery disease
CCC: Concordance correlation coefficient
CS: Circumferential strain
E: Early mitral inflow velocity
e′: Early diastolic mitral annular tissue velocity
EF: Ejection fraction
GLS: Global longitudinal strain
HsCRP: High-sensitivity C-reactive protein
ICC: Interclass correlation
LV: Left ventricular
LVEF: Left ventricular ejection fraction
MESA: Multi-Ethnic Study of Atherosclerosis
OR: Odds ratio
ROC: Receiver operating coefficient
S: Strain
SR: Systolic strain rate
TTP: Time-to-peak
VVI: Velocity vector imaging
